# DRA involvement in linaclotide-stimulated bicarbonate secretion during loss of CFTR function

**DOI:** 10.1172/jci.insight.172364

**Published:** 2024-06-13

**Authors:** Jessica B. Sarthi, Annie M. Trumbull, Shayda M. Abazari, Vincent van Unen, Joshua E. Chan, Yanfen Jiang, Jesse Gammons, Marc O. Anderson, Onur Cil, Calvin J. Kuo, Zachary M. Sellers

**Affiliations:** 1 Department of Pediatrics, Division of Gastroenterology, Hepatology, and Nutrition; and; 2 Department of Medicine, Division of Hematology, Stanford University, Palo Alto, California, USA.; 3 Department of Chemistry and Biochemistry, San Francisco State University, San Francisco, California, USA.; 4 Department of Pediatrics, University of California, San Francisco, San Francisco, California, USA.; 5 Sellers Research and Clinical Development, LLC, Newark, California, USA.

**Keywords:** Gastroenterology, Epithelial transport of ions and water, Guanylate cyclase

## Abstract

Duodenal bicarbonate secretion is critical to epithelial protection, as well as nutrient digestion and absorption, and is impaired in cystic fibrosis (CF). We examined if linaclotide, typically used to treat constipation, may also stimulate duodenal bicarbonate secretion. Bicarbonate secretion was measured in vivo and in vitro using mouse and human duodenum (biopsies and enteroids). Ion transporter localization was identified with confocal microscopy, and de novo analysis of human duodenal single-cell RNA sequencing (scRNA-Seq) data sets was performed. Linaclotide increased bicarbonate secretion in mouse and human duodenum in the absence of cystic fibrosis transmembrane conductance regulator (CFTR) expression (*Cftr*-knockout mice) or function (CFTR_inh_-172). Na^+^/H^+^ exchanger 3 inhibition contributed to a portion of this response. Linaclotide-stimulated bicarbonate secretion was eliminated by down-regulated in adenoma (DRA, *SLC26A3*) inhibition during loss of CFTR activity. ScRNA-Seq identified that 70% of villus cells expressed *SLC26A3*, but not *CFTR*, mRNA. Loss of CFTR activity and linaclotide increased apical brush border expression of DRA in non-CF and CF differentiated enteroids. These data provide further insights into the action of linaclotide and how DRA may compensate for loss of CFTR in regulating luminal pH. Linaclotide may be a useful therapy for CF individuals with impaired bicarbonate secretion.

## Abstract 2

Linaclotide stimulates duodenal bicarbonate secretion upon loss of CFTR function by increasing membrane trafficking and activity of DRA, indicating potential therapeutic value in cystic fibrosis.

## Graphical abstract

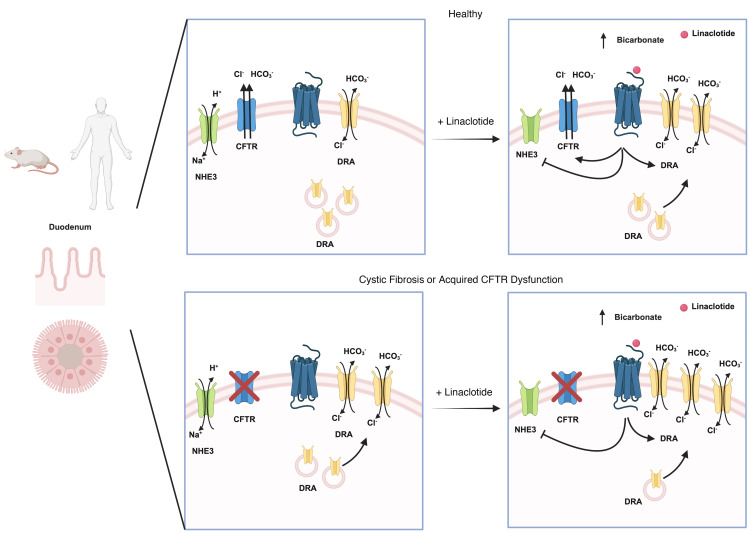

## Introduction

Proximal duodenal bicarbonate secretion, together with pancreatic bicarbonate secretion, is integral to preventing intestinal mucosal injury from gastric secretions and establishing a nonacidic intraluminal environment for activation of digestive enzymes. This importance is highlighted by cystic fibrosis (CF) intestinal disease and malnutrition, where decreased bicarbonate secretion is central to disease pathogenesis (1, 2). Duodenal bicarbonate secretion is highly regulated, driven by luminal acid and neurohormonal signaling, ultimately resulting in coordinated transepithelial bicarbonate secretion by the cystic fibrosis transmembrane conductance regulator (CFTR), chloride/bicarbonate exchange (e.g., down-regulated in adenoma, DRA; putative anion transporter-1, PAT-1), or decreased proton transport through Na^+^/H^+^ exchange (e.g., NHE3) (3). Despite its physiologic importance, there are limited therapies targeting duodenal bicarbonate secretion.

Linaclotide is an FDA-approved medication for the treatment of constipation-predominant irritable bowel syndrome and chronic idiopathic constipation whose effect relies on its structural similarity to the heat-stable enterotoxin of *Escherichia coli* (STa). Both STa and linaclotide bind guanylyl cyclase C (GC-C) receptors on the apical surface of enterocytes and cause increases in CFTR-dependent chloride secretion and inhibition of NHE3-mediated sodium absorption (4–6). Previously, we identified that STa stimulates duodenal bicarbonate secretion through CFTR and CFTR-independent means (7, 8). Linaclotide has been investigated as a potential therapy for CF-related constipation (6, 9, 10); however, it is unclear if linaclotide stimulates duodenal bicarbonate secretion and if linaclotide could improve impaired intraluminal pH in patients with CF by stimulating CFTR-independent bicarbonate secretion.

We undertook a series of experiments using in vivo and in vitro bicarbonate secretion measurements in mice and humans to examine the effect of linaclotide on duodenal bicarbonate secretion. We coupled these experiments with scRNA-Seq analysis of acid-based transporters in the human duodenum and confocal microscopy to characterize the brush border expression of the DRA chloride/bicarbonate exchanger in human duodenum in the presence or absence of CFTR activity and/or expression.

## Results

### Linaclotide stimulates bicarbonate secretion in mouse and human duodenum.

**Figure 1:**
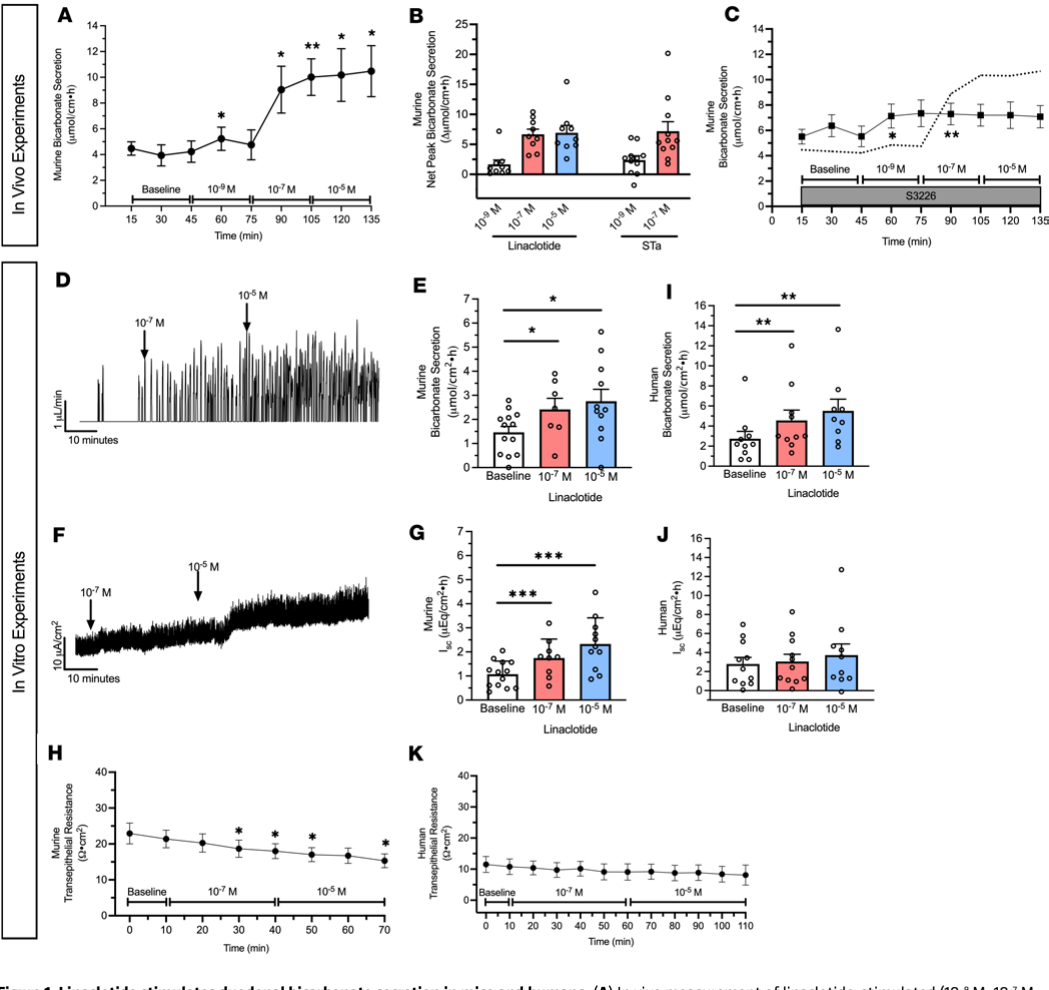
Linaclotide stimulates duodenal bicarbonate secretion in mice and humans. (**A**) In vivo measurement of linaclotide-stimulated (10^–9^ M, 10^–7^ M, 10^–5^ M) duodenal bicarbonate secretion in C57BL6/J mice (*n* = 9). After 30-minute baseline measurements, each linaclotide dose was sequentially perfused for 30 minutes. *P < 0.05; **P < 0.01 vs. baseline by 1-way ANOVA. (**B**) Net peak (max – baseline) bicarbonate secretion. Linaclotide values were from **A**. For STa (10^–9^ M, 10^–7^ M), separate experiments were performed (*n* = 11). Each point represents a different mouse. (**C**) In vivo effect of linaclotide (10^–9^ M, 10^–7^ M, 10^–5^ M) with NHE3 inhibitor S3226 (10^–5^ M) (*n* = 8). Dotted line is the mean response to linaclotide (from **A**). *P < 0.05; **P < 0.01 vs. baseline by 1-way ANOVA. (**D**) Representative trace for in vitro bicarbonate secretion in mouse duodenum. *Y* axis is titrated HCl, which reflects base secreted by the tissue. During baseline there are few peaks. Upon stimulation, the number of titration events and/or volume of titrant increases. (**E**) In vitro baseline and linaclotide-stimulated (10^–7^, 10^–5^ M, apical) mouse duodenal mucosal bicarbonate secretion. Each point (*n* = 8–13) is a separate piece of duodenum from 10 mice. *P < 0.05 vs. baseline by 1-way ANOVA. (**F**) Representative short-circuit current (I_sc_) trace for in vitro mouse experiments. (**G**) In vitro baseline and linaclotide-stimulated (10^–7^, 10^–5^ M, apical) mouse duodenal I_sc_. Each point represents a separate piece of duodenum from the same mice in **E**. ***P < 0.001 vs. baseline by 1-way ANOVA. (**H**) In vitro transepithelial resistance measurements from **E** and **G**. *P < 0.05 vs. baseline by 1-way ANOVA. (**I**–**K**) Human duodenal mucosal bicarbonate secretion (**I**), I_sc_ (**J**), and transepithelial resistance (**K**) from endoscopic biopsies. Experiments were performed and data expressed similar as murine experiments in **D**–**H**. Each point (*n* = 9–10) represents a different biopsy. **P < 0.01 vs. baseline by 1-way ANOVA. All data are means ± SEM, unless stated otherwise.

To determine linaclotide’s ability to stimulate intestinal bicarbonate secretion, we examined its effect on the duodenal segment of the small intestine, given the robust expression of acid-base transporters in this segment that function to neutralize the highly acidic gastric contents it encounters. We first examined the effect of in vivo intestinal perfusion of linaclotide on duodenal bicarbonate secretion. Linaclotide stimulated sustained increases in bicarbonate secretion at 10^–7^ M and 10^–5^ M (*P* < 0.001 and *P* = 0.004, *n* = 9), with small and transient increases in bicarbonate secretion over baseline secretory rates at 10^–9^ M (*P* = 0.033, *n* = 9). There was no difference between 10^–7^ M or 10^–5^ M (*P* = 0.926) (Figure 1, A and B), and bicarbonate secretory rates were similar to those observed with STa (Figure 1B, 10^–9^ M: *P* = 0.491 and 10^–7^ M: 0.792, respectively, *n* = 11). Linaclotide did not stimulate net increases in luminal duodenal fluid secretion (10^–9^ M: mean ± SEM 0.01 ± 0.05, 10^–7^ M: –0.05 ± 0.06, 10^–5^ M: –0.04 ± 0.05 ΔmL/cm·h, *P* = 0.856, 0.445, 0.367, respectively, *n* = 8), similar to what we have previously reported for STa in the duodenum (7). As linaclotide can decrease proton secretion by inhibiting NHE3 (6, 9), we examined if linaclotide actively stimulates bicarbonate secretion or purely inhibits baseline proton secretion to raise intraluminal pH. Using the same in vivo technique, perfusion with S3226 (10^–5^ M), a selective inhibitor of NHE3, caused a significant increase in baseline bicarbonate secretion (3.78 ± 0.73 vs. 6.70 ± 0.68 μmol/cm·h, *n* = 9–10, *P* = 0.016). In the presence of S3226, linaclotide caused further increases in bicarbonate secretion (10^–9^ M: 2.22 ± 0.87, 10^–7^ M: 2.53 ± 0.82, 10^–5^ M: 2.38 ± 1.32 Δμmol/cm·h, *n* = 10, *P* = 0.075, *P* = 0.032, *P* = 0.237, respectively) (Figure 1C). In the presence of S3226, linaclotide-stimulated duodenal bicarbonate secretion was approximately one-third of the response without S3226 (10^–7^ M: 37.9% ± 12.3%, 10^–5^ M: 35.6% ± 19.8%). Thus, in mouse duodenum, linaclotide alters duodenal luminal pH through a combination of inhibiting NHE3-mediating proton secretion and stimulating bicarbonate secretion.

To further characterize linaclotide-stimulated bicarbonate secretion, we performed combined short-circuit current (I_sc_) and bicarbonate secretory measurements in mouse duodenum stripped of muscle and serosa and mounted in Ussing chambers. As seen in Figure 1, D–G, this in vitro method showed similar results as the in vivo method. Linaclotide stimulated significant increases in duodenal bicarbonate secretion at 10^–7^ M (*P* = 0.025, *n* = 9) and 10^–5^ M (*P* = 0.047, *n* = 11), with no difference between them (*P* = 0.875). I_sc_ analysis revealed that linaclotide stimulated significant increases in I_sc_ at 10^–7^ M (*P* < 0.001, *n* = 9) and 10^–5^ M (*P* < 0.001, *n* = 11) (Figure 1, F and G). Bicarbonate secretion and I_sc_ were accompanied by small, but significant (*P* < 0.05), changes in transepithelial resistance (Figure 1H).

We next examined the potential for linaclotide to stimulate bicarbonate secretion in human duodenum using endoscopically obtained duodenal biopsies from people without known acid-base disturbances or histologic evidence of duodenal disease. Biopsies were mounted in Ussing chambers, and combined bicarbonate secretion and I_sc_ measurements were performed, similar to Figure 1, D–H. As seen in Figure 1I, 10^–7^ M and 10^–5^ M linaclotide stimulated significant increases in human duodenal bicarbonate secretion (*P* = 0.006, *n* = 11; and *P* = 0.004, *n* = 10, respectively), at similar magnitudes previously reported for STa in human duodenal biopsies (11). In contrast with mice, linaclotide did not stimulate a change in I_sc_ in human biopsies (10^–7^ M: *P* = 0.071, *n* = 11; 10^–5^ M: *P* = 0.186, *n* = 10) (Figure 1J) or transepithelial resistance (Figure 1, J and K). Thus, linaclotide stimulates electrogenic bicarbonate secretion in mouse duodenum and electroneutral bicarbonate secretion in human duodenum.

### Linaclotide-stimulated duodenal bicarbonate secretion occurs independent of CFTR expression or function.

**Figure 2:**
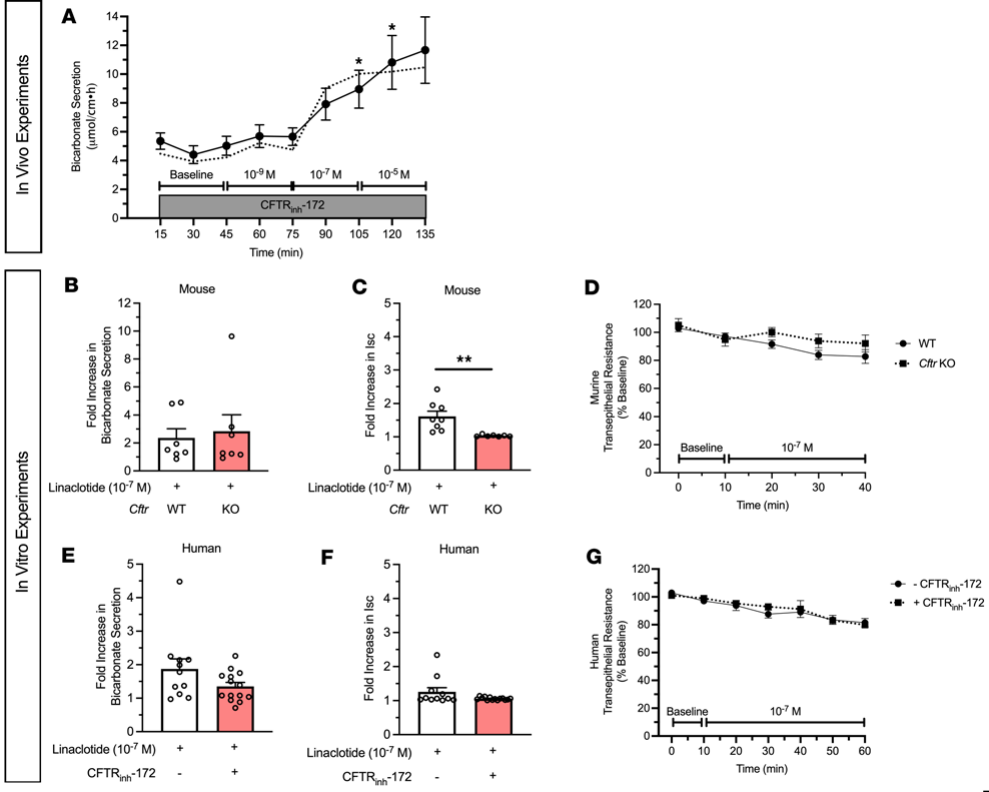
Linaclotide stimulates duodenal bicarbonate secretion independent of CFTR. (**A**) In vivo measurement of duodenal bicarbonate secretion in mice, similar to Figure 1A, with the exception that each perfusate also contained CFTR_inh_-172 (2 × 10^–5^ M) (*n* = 12). Dotted line is the mean response without CFTR_inh_-172 (from Figure 1A). *P < 0.05 vs. baseline by 1-way ANOVA. (**B** and **C**) In vitro duodenal mucosal bicarbonate secretion (**B**) and I_sc_ (**C**) in wild-type (WT) and *Cftr*-KO mice. Data are expressed as fold increase in linaclotide-stimulated responses over baseline bicarbonate secretion or I_sc_ in the same mouse. Each point (*n* = 7) is a separate piece of duodenum from 5 mice. **P < 0.01 by unpaired 2-tailed Student’s *t* test. (**D**) Transepithelial resistance measurements in WT and *Cftr-*KO mice. (**E**–**G**) In vitro duodenal mucosal bicarbonate secretion (**E**), I_sc_ (**F**), and transepithelial resistance (**G**) in human endoscopic biopsies with or without CFTR_inh_-172 pretreatment (2 × 10^–5^ M, 40–60 minutes). Each point (*n* = 11–14) represents a different biopsy. All data are means ± SEM.

Next, given our prior findings that STa can stimulate CFTR-independent duodenal bicarbonate secretion in mice (7), we studied linaclotide’s dependence on CFTR for bicarbonate secretion. We first repeated the in vivo bicarbonate secretion experiments by perfusing the duodenum with CFTR_inh_-172, a specific CFTR inhibitor (12). Linaclotide continued to stimulate significant bicarbonate secretory responses at 10^–7^ M and 10^–5^ M (*P* = 0.008 and *P* = 0.018, respectively, *n* = 12), but not 10^–9^ M (*P* = 0.198, *n* = 12), in the presence of CFTR_inh_-172 (2 × 10^–5^ M) (Figure 2A). In comparison, in vivo, CFTR_inh_-172 inhibited forskolin-stimulated (10^–4^ M) bicarbonate secretion by 65.1% ± 12.5% (*P* = 0.022), preventing increases over baseline (*P* = 0.778, *n* = 6–9) (Supplemental Figure 1, A and B; supplemental material available online with this article; https://doi.org/10.1172/jci.insight.172364DS1). In vitro, sequential addition of CFTR inhibitors with different mechanisms of action, GlyH-101 (10^–5^ M, mucosal) and glibenclamide (3 × 10^–4^ M, mucosal), following CFTR_inh_-172 (2 × 10^–5^ M, serosal), resulted in no further increase in the inhibition of forskolin-stimulated I_sc_ (0.2% and –0.9%, respectively). To expand upon these pharmacologic experiments, we performed in vitro Ussing chamber experiments with *Cftr*^tm1UNC^-knockout (KO) mice, which have global KO of the *Cftr* gene (13). Given the similar responses between 10^–7^ M and 10^–5^ M in prior experiments, for these studies we specifically focused on 10^–7^ M linaclotide. We found that linaclotide (10^–7^ M) stimulated significant increases in duodenal bicarbonate secretion in *Cftr*-KO mice (*P* = 0.028 vs. baseline, *n* = 7). These magnitudes were similar to wild-type (WT) mice (*P* = 0.728) (Figure 2B). In contrast with mice expressing CFTR, linaclotide stimulated minimal change in I_sc_ in *Cftr*-KO mice (0.77 ± 0.31 ΔμA/cm^2^, *P* = 0.049, *n* = 8) with no change in transepithelial resistance (*P* = 0.396) (Figure 2, C and D), suggesting that in the absence of CFTR, linaclotide stimulates electroneutral bicarbonate secretion. To determine if these findings hold true in humans, we pretreated human endoscopic duodenal biopsies with CFTR_inh_-172, which inhibits forskolin-stimulated I_sc_ in human duodenal enteroids (14) (Supplemental Figure 1C), and then performed repeat Ussing chamber measurements. Linaclotide (10^–7^ M) continued to stimulate significant increases in duodenal bicarbonate secretion in biopsies pretreated with CFTR_inh_-172 (2 × 10^–5^ M, serosal, *P* = 0.024, *n* = 14), at a magnitude that was comparable to biopsies without CFTR_inh_-172 treatment (*P* = 0.644) (Figure 2E). There was no difference in linaclotide-stimulated I_sc_ with or without CFTR_inh_-172 treatment (0.27 ± 0.11 vs. 0.13 ± 0.04 ΔμEq/cm^2^·h, *P* = 0.239, *n* = 11–14) (Figure 2F). Transepithelial resistance in the presence of CFTR_inh_-172 was unchanged before or after linaclotide stimulation (*P* = 0.889, *n* = 14) (Figure 2G).

### Linaclotide-stimulated duodenal bicarbonate secretion upon loss of CFTR function is mediated by the DRA chloride/bicarbonate exchanger.

**Figure 3:**
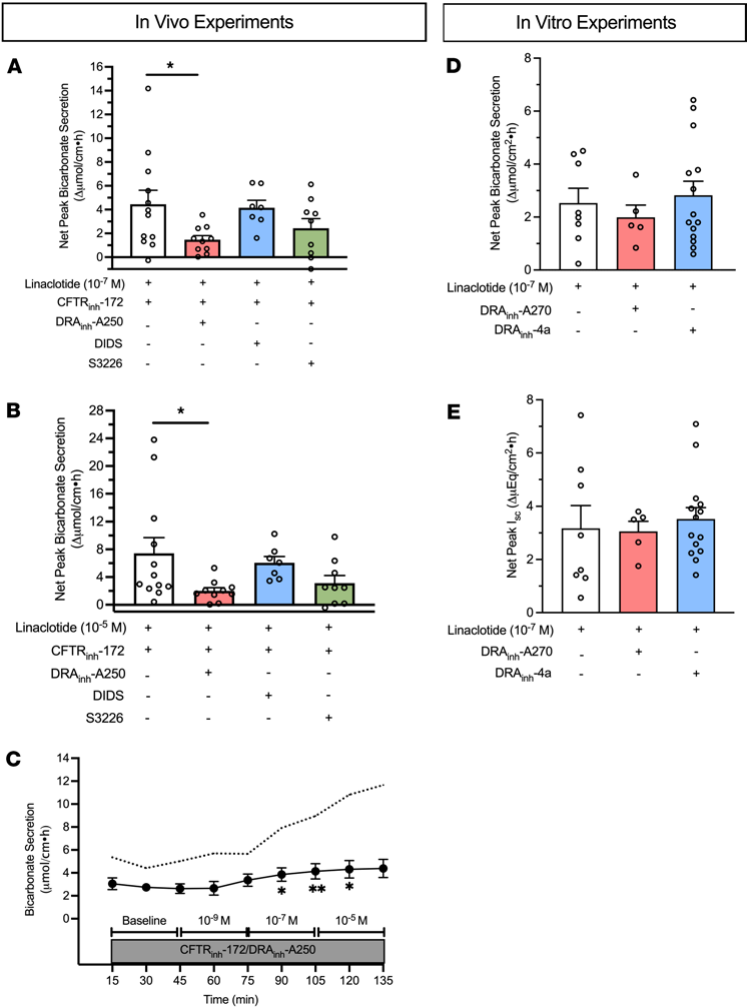
Key role of DRA in CFTR-independent linaclotide-stimulated duodenal bicarbonate secretion upon loss of CFTR. (**A** and **B**)To determine the source of linaclotide-stimulated bicarbonate transport in the absence of CFTR function, in vivo experiments were repeated, similar to Figure 2A, except in addition to CFTR_inh_-172 (2 × 10^–5^ M, *n* = 12), one of the following was also added to the luminal perfusate: DRA_inh_-A250 (10^–5^ M, *n* = 10), DIDS (2 × 10^–4^ M, *n* = 7), or S3226 (10^–5^ M, *n* = 9). (**A**) Linaclotide 10^–7^ M, (**B**) linaclotide 10^–5^ M. Each point represents a different mouse. *P < 0.05 vs. linaclotide + CFTR_inh_-172 (2 × 10^–5^ M) by 1-way ANOVA. (**C**) Time course with linaclotide dose response (10^–9^ M, 10^–7^ M, 10^–5^ M) in the presence of CFTR_inh_-172 (2 × 10^–5^ M) and DRA_inh_-A250 (10^–5^ M), as indicated by circles and whiskers (*n* = 12). Dotted line indicates mean response in the presence of CFTR_inh_-172 (2 × 10^–5^ M) only (from Figure 2A). *P < 0.05; **P < 0.01 vs. baseline by 1-way ANOVA. (**D**) Net peak linaclotide-stimulated (10^–7^ M, apical) mouse duodenal mucosal bicarbonate secretion (**D**) and I_sc_ (**E**) from in vitro experiments, with or without DRA inhibition by DRA_inh_-A270 (10^–5^ M, bilateral, *n* = 5) or DRA_inh_-4a (10^–5^ M, bilateral, *n* = 14). Each point represents a separate piece of duodenum from 5–10 mice. All data are means ± SEM.

To investigate the potential mechanism whereby linaclotide increases duodenal bicarbonate secretion independent of CFTR, we investigated the effect of DRA_inh_-A250 (selective inhibitor of DRA), 4,4′-Diisothiocyanatostilbene-2,2′-disulfonate (DIDS; DRA-insensitive, nonselective anion exchanger inhibitor), and S3226 (selective inhibitor of NHE3) on duodenal bicarbonate secretion. Using our in vivo model, in separate experiments, each drug was combined with CFTR_inh_-172 and perfused prior to stimulation with linaclotide. DRA_inh_-A250 (10^–5^ M) caused a 67% ± 8% reduction in CFTR-independent, linaclotide-stimulated duodenal bicarbonate secretion at 10^–7^ M linaclotide (*P* = 0.044, *n* = 10) and 73% ± 6% inhibition at 10^–5^ M linaclotide (*P* = 0.038, *n* = 10) (Figure 3, A–C). In contrast, DIDS (2 × 10^–4^ M) failed to affect CFTR-independent linaclotide-stimulated duodenal bicarbonate secretion (10^–7^ M: –7% ± 14%, *P* = 0.993, *n* = 7; 10^–5^ M: –19% ± 14%, *P* = 0.893, *n* = 7). S3226 (10^–5^ M) reduced CFTR-independent linaclotide-stimulated duodenal bicarbonate secretion by 45% ± 19% (10^–7^ M) and 58% ± 16% (10^–5^ M); however, neither of these was statistically significant (*P* = 0.254, *P* = 0.136, respectively, *n* = 9) (Figure 3, A and B). Therefore, it appears that DRA is the primary source of bicarbonate transport for CFTR-independent, linaclotide-stimulated duodenal bicarbonate secretion.

To determine if DRA’s critical role in linaclotide-stimulated duodenal bicarbonate secretion is unique to when there is functional loss of CFTR, we examined the role of DRA in mouse duodenal bicarbonate secretion measured in vitro. For these experiments, DRA_inh_-A270, a nearly identical chemical compound as DRA_inh_-A250 (Br^–^ to I^–^ substitution) with similar DRA specificity and inhibitory properties as DRA_inh_-A250 at 10^–5^ M (15), was used. DRA_inh_-A270 (10^–5^ M) had no inhibitory impact on linaclotide-stimulated duodenal bicarbonate secretion (*P* = 0.813, *n* = 5–8) or I_sc_ (*P* = 0.991, *n* = 5–8) in the presence of CFTR function (Figure 3, D and E). In vivo testing also showed no inhibitory effect of DRA_inh_-A270 on linaclotide-stimulated bicarbonate secretion without CFTR inhibition (DMSO + linaclotide 10^–7^ M: 4.3 ± 1.1 vs. DRA_inh_-A270 + linaclotide 10^–7^ M: 3.5 ± 0.3 µmol/cm•h, *n* = 4 each, *P* = 0.542). To verify lack of effect of DRA inhibition under these conditions, we performed additional in vitro experiments using DRA_inh_-4a, a DRA-specific inhibitor that is structurally and mechanistically distinct from DRA_inh_-A250 and DRA_inh_-A270 (16). These experiments showed similar results as DRA_inh_-A270: no inhibition of linaclotide-stimulated duodenal bicarbonate secretion (*P* = 0.899, *n* = 8–16) or I_sc_ (*P* = 0.870, *n* = 8–16) in the presence of CFTR function. Thus, CFTR is sufficient for linaclotide-stimulated bicarbonate secretion, but upon loss of CFTR function, DRA appears to compensate for its loss.

### DRA expression in human duodenum.

**Figure 4:**
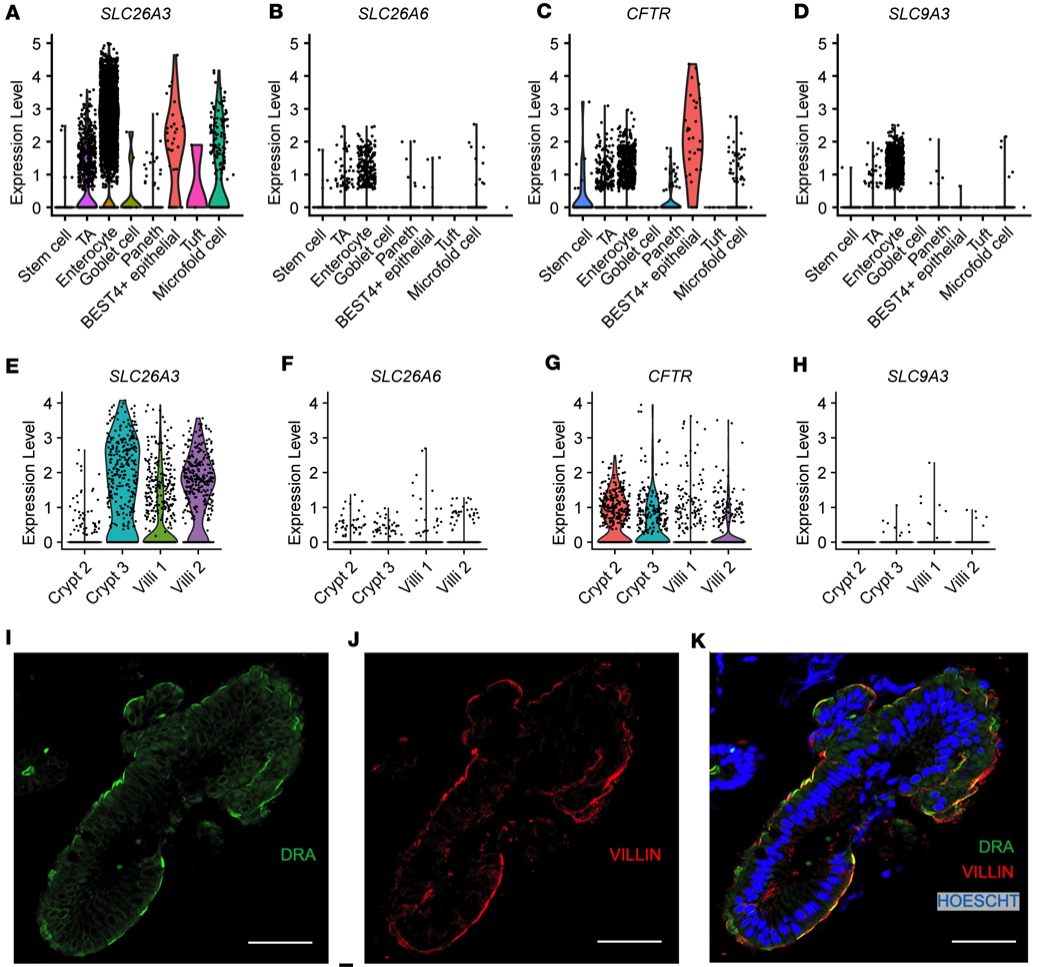
Cellular and membrane expression of *SLC26A3* (DRA) in human duodenal enterocytes. (**A**–**H**) Cellular mRNA expression of *SLC26A3* (DRA), *SLC26A6* (PAT-1), *CFTR*, and *SLC9A3* (NHE3) based on reanalysis of Elmentaite et al. (21) (**A**–**D**) and Busslinger et al. (20) (**E**–**H**). Violin plots represent expression relative to all cells of that type, with each point representing the expression of individual cells within each type. TA, transit-amplifying cells. (**I**–**K**) Representative confocal immunofluorescence imaging of DRA (**I**), villin (marker of apical brush border, **J**), and nucleus (**K**) in human duodenum from endoscopic biopsy (*n* = 3). Scale bar = 20 μm.

DRA and PAT-1 are the primary chloride/bicarbonate exchangers expressed in mouse intestine, with PAT-1 showing dominant RNA expression in the small intestine and DRA RNA being more abundant in the colon (17). There are limited data regarding DRA expression in human duodenum (18, 19). We capitalized on 2 previously published human duodenum single-cell RNA-Seq (scRNA-Seq) data sets (20, 21) to examine DRA mRNA expression in human duodenum. The Elmentaite et al. data set (21) contained 5,944 cells with an average of 4,164 reads per cell from 5 healthy adult duodenal samples, allowing for high confidence in cell type separation. The Busslinger et al. data set (20) contained 702 crypt cells and 923 villus cells from 2 adult duodenal samples, with an average of 10,381 reads per cell. Data from 1 crypt sample (Crypt 1) were excluded due to very low sequence reads (2,088 per cell). We verified accurate classification into crypt and villus cells by examining *LGR5* expression and proliferation markers *MKI67* and *PCNA*, which are generally restricted to the crypt region. These were all more abundant in the crypt group, with *LGR5* being exclusively expressed in the crypt, but not villus, group (Supplemental Figure 2, A–C). Using these 2 data sets, we examined expression of the key acid-base transporters in the duodenum, DRA (*SLC26A3*), PAT-1 (*SLC26A6*), *CFTR*, and NHE3 (*SLC9A3*). Our analysis showed that *SLC26A3* mRNA was predominantly expressed in enterocytes (Figure 4A), in both the crypt and villus groups (Figure 4E), with higher expression and expression in more cells than *SLC26A6*, *CFTR*, or *SLC9A3* (Figure 4, A–H). Of note, Crypt 2 had lower *SLC26A3* expression than Crypt 3, which correlated with higher *CFTR*, *MKI67*, and *PCNA* expression; more *LGR5*^+^ cells; and fewer *SLC9A3*^+^ cells, suggesting Crypt 2 contained more cells deeper within the crypts than Crypt 3 (Figure 4, E, G, and H, and Supplemental Figure 2). We verified DRA protein expression in human duodenal biopsies (*n* = 3) using immunofluorescence imaging. The majority of DRA is localized to the intracellular apical region, with a smaller fraction present at the apical brush border (Figure 4, I–K).

**Figure 5:**
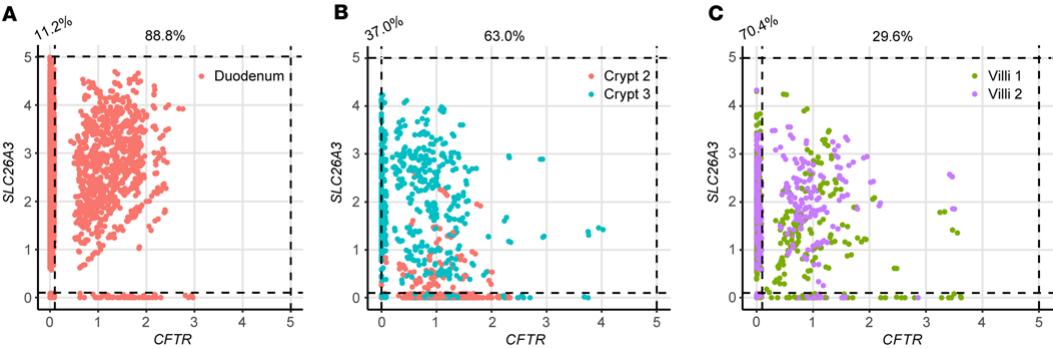
*SLC26A3* and *CFTR* coexpression in human duodenum by scRNA-Seq. (**A**–**C**) Coexpression of *SLC26A3* (DRA) and *CFTR* mRNA using FeatureScatter based on Elmentaite et al. (21) enterocytes (**A**) and Busslinger et al. (20) crypt and villi (**B** and **C**) data sets that were analyzed in Figure 4. Numbers on top of graphs represent the percentage of *SLC26A3*-expressing cells that express *SLC26A3* only (left) or *SLC26A3* and *CFTR* (right).

Given DRA’s functional independence from CFTR in mediating linaclotide-stimulated duodenal bicarbonate secretion, we quantified the proportion of *SLC26A3*-expressing cells that coexpressed *CFTR*. In the Elmentaite et al. data set, 88.8% of *SLC26A3*-expressing enterocytes coexpressed *SLC26A3* and *CFTR* (Figure 5A). Using the Busslinger et al. data set to examine crypt versus villus localization, 63.0% of *SLC26A3*-positive crypt cells coexpressed *SLC26A3* and *CFTR*, whereas in the villus cells, only 29.6% did so (Figure 5, B and C). These findings were not ubiquitous for all chloride/bicarbonate exchangers. *SLC26A6* and *CFTR* coexpression was relatively low in enterocytes (16.7%) (Supplemental Figure 3A), but examination of the crypt and villus cell types showed a higher percentage of *CFTR* coexpression with *SLC26A6* than *SLC26A3*, with 81.8% of crypt cells and 42.9% of villus cells coexpressing *SLC26A6* and *CFTR* (Supplemental Figure 3, B and C). In the Elmentaite et al. data set, *SLC9A3* and *CFTR* were coexpressed in 14.3% of enterocytes, similar to *SLC26A6* (Supplemental Figure 3D). The Busslinger et al. data set possessed insufficient numbers of *SLC9A3*-expressing cells to analyze *SLC9A3* and *CFTR* coexpression in crypts and villi.

### Linaclotide alters the apical localization of DRA.

**Figure 6:**
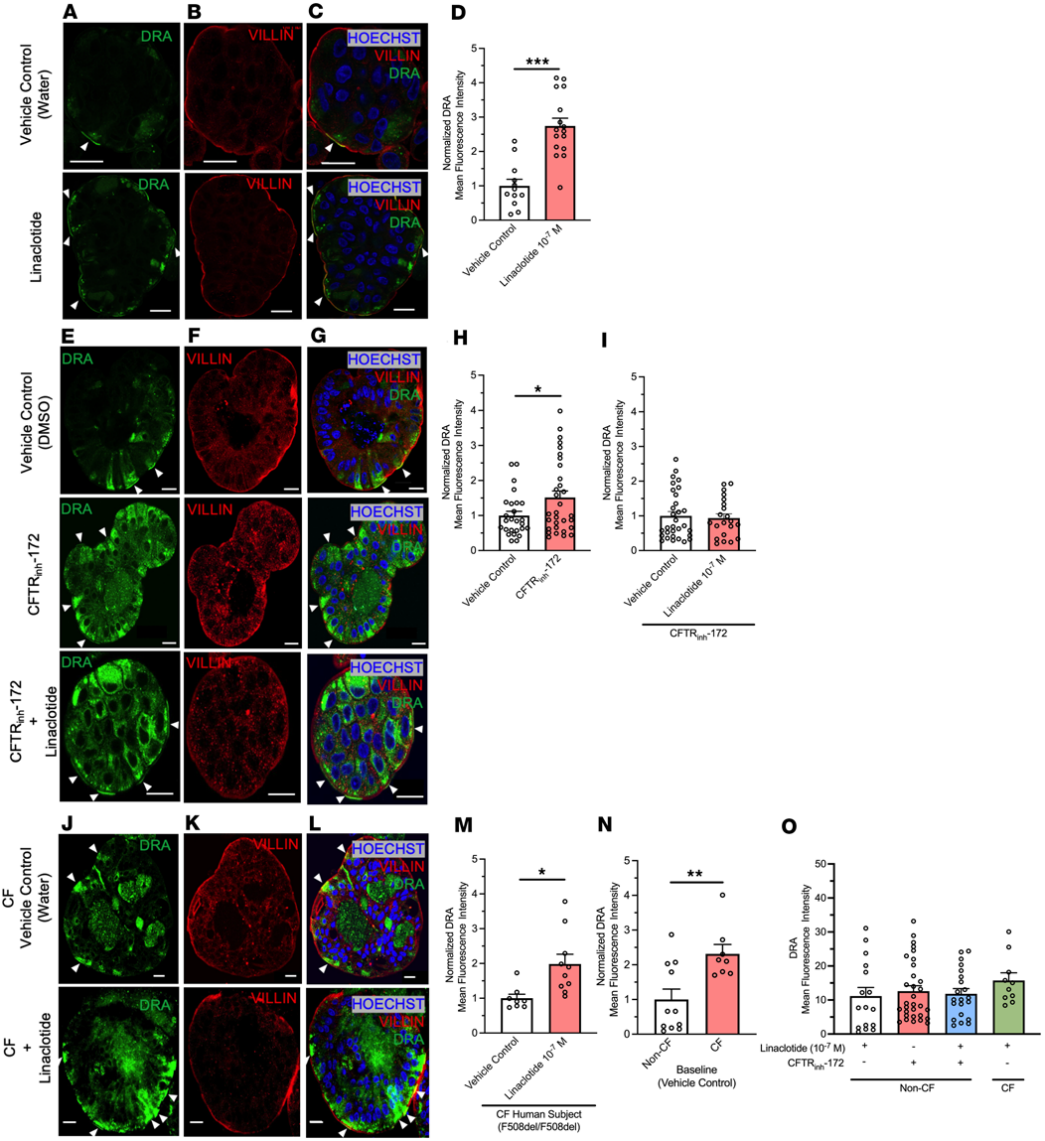
Linaclotide increases membrane expression of DRA in apical-out human duodenal enteroids. Representative images of DRA (**A** and **E**), villin (**B** and **F**), and DRA and villin and Hoechst (**C** and **G**) in apical-out healthy duodenal enteroids during control conditions (water or DMSO), CFTR_inh_-172 (2 × 10^–5^ M), or linaclotide (10^–7^ M) with or without CFTR_inh_-172 pretreatment (2 × 10^–5^ M) (all 40 minutes). (**D**, **H**, and **I**) Quantification of DRA at the apical brush border using villin to define this cell region. Apical brush border DRA MFI following linaclotide (10^–7^ M, 40 minutes, *n* = 16), CFTR_inh_-172 alone (2 × 10^–5^ M, 40 minutes, *n* = 31), or CFTR_inh_-172+linaclotide (*n* = 21). Treatments were normalized to vehicle controls (water for linaclotide, DMSO for CFTR_inh_-172, *n* = 12–31). Enteroids from 3 individuals were used for each condition. Significance determined by unpaired, 2-tailed Student’s *t* test. *P < 0.05, ***P < 0.001. Representative images of DRA (**J**), villin (**K**), and DRA and villin and Hoechst (**L**) in apical-out CF (F508del homozygous) duodenal enteroids during control (water, 40 minutes) or linaclotide stimulation (10^–7^ M, 40 minutes). (**M**) Quantification of DRA present at the apical brush border using villin, similar to **D**. (**N**) Comparison of baseline DRA MFI between non-CF and CF enteroids, with data normalized to non-CF enteroids. *n* = 8–11 enteroids from 1 patient across 2 different passages. Significance determined by unpaired, 2-tailed Student’s *t* test. *P < 0.05, **P < 0.01. (**O**) To compare DRA membrane expression across non-CF and CF enteroids in different situations, absolute DRA membrane MFI was plotted. Data are from experiments performed in **D**, **H**, **I**, and **M**. In images, arrows assist with identifying regions of interest (scale bar, 20 µm). Columns with whiskers are mean ± SEM with each dot representing a different enteroid.

Ion channel activity can be regulated by altering membrane expression of channels. We examined if linaclotide alters DRA activity by changing its apical brush border expression. For these experiments we used human derived 3-dimensional duodenal enteroids. Given that the linaclotide receptor GC-C is expressed at the apical surface, we generated reverse-polarity apical-out duodenal enteroids, which allow facile access to the apical membrane via the enteroid exterior (Supplemental Figure 4, A and B) (22). Using quantitative PCR (qPCR), we verified that apical-out enteroids express *CFTR*, *SLC26A3*, *SLC26A6*, *SLC9A3*, and *GUCY2C* in undifferentiated and 3-day differentiated enteroids (Supplemental Figure 4, C and D). As expected, *LGR5* was decreased in differentiated apical-out enteroids. Using this model of human duodenum, we incubated apical-out differentiated enteroids with linaclotide (10^–7^ M, 40 minutes) or vehicle control (water, 40 minutes) and performed confocal immunofluorescence to localize DRA. As seen in Figure 6, A–D, linaclotide significantly increased apical brush border expression of DRA (*n* = 12–16). In contrast, when we performed the same experiment with NHE3 localization, we found no change in the apical brush border expression of NHE3 upon linaclotide stimulation (Supplemental Figure 5, A–D). We verified that myosin VI (*MYO6*), a key protein in NHE3 trafficking (23), mRNA was present in apical-out enteroids. *MYO6* mRNA was expressed at similar levels in this model as many of the other transporters we examined (Supplemental Figure 4, C and D). To determine if linaclotide also stimulates DRA localization upon lack of functional CFTR, we repeated the experiments in the presence of CFTR_inh_-172 (2 × 10^–5^ M, 40 minutes). Compared with vehicle control (DMSO, 40 minutes), CFTR inhibition resulted in an increase in the apical expression of DRA (*P* = 0.032, *n* = 26–31) (Figure 6, E–H) but not NHE3 (*P* = 0.633, *n* = 16–30) (Supplemental Figure 5, E–H). Of note, DMSO alone increased DRA brush border expression compared with water (MFI means ± SEM: 10.94 ± 1.32 vs. 3.43 ± 1.03, *n* = 11–15, *P* < 0.001). Linaclotide stimulation in the presence of CFTR_inh_-172 did not alter DRA or NHE3 brush border expression (*P* = 0.730, *n* = 31; *P* = 0.535, *n* = 29–30, respectively) (Figure 6, E–G and I, and Supplemental Figure 5, E–G and I).

To determine how our CFTR_inh_-172 studies compare to chronic loss of CFTR expression and function, we examined DRA brush border expression in human duodenal enteroids isolated from a CF individual with F508del homozygous *CFTR* variants. Comparison of baseline (water vehicle control, 40 minutes) DRA brush border expression between non-CF and CF enteroids showed CF enteroids had increased DRA expression at the apical brush border (Figure 6, J–M, n = 8–11, *P* = 0.006). Linaclotide stimulation (10^–7^ M, 40 minutes) increased DRA brush border expression in F508del homozygous CF enteroids, compared with CF enteroids treated with vehicle control (water, 40 minutes) (Figure 6, J–L and N, n = 8–10, *P* = 0.010). To examine the discrepancies between CFTR_inh_-172 + linaclotide in non-CF enteroids and linaclotide in the CF enteroids, we examined the absolute MFIs of each condition. We found that CFTR_inh_-172 alone caused similar increases in absolute DRA brush border expression as linaclotide in non-CF enteroids (with or without CFTR_inh_-172) or CF enteroids (Figure 6O, n = 10–31, *P* = 0.676), suggesting that lack of additional DRA membrane expression in non-CF enteroids treated with CFTR_inh_-172 + linaclotide may be due to maximal DRA brush border expression already being achieved upon CFTR_inh_-172 treatment. Taken together with our bicarbonate secretory measurements, these data show that linaclotide can alter DRA activity and/or brush border expression, in the presence or absence of functional CFTR. In contrast, linaclotide appears to modulate NHE3 activity, rather than brush border expression, regardless of CFTR function.

### Inhibition of CFTR transiently increases intracellular pH.

**Figure 7:**
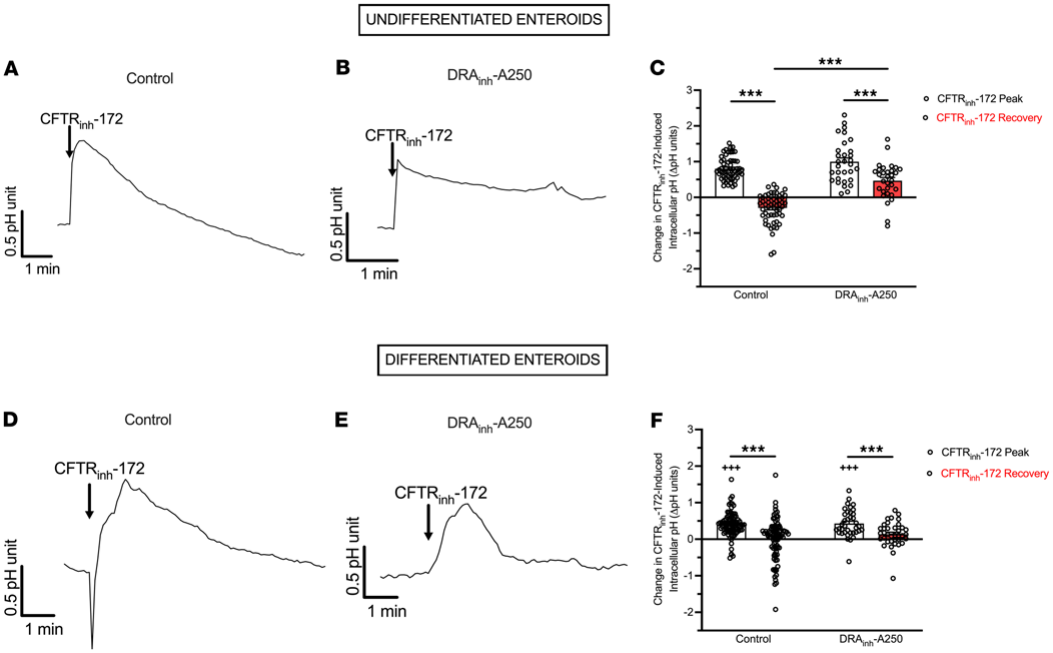
Inhibition of CFTR activity induces increases in pH_i_. (**A** and **B**) Mean time course of CFTR_inh_-172–induced (2 × 10^–5^ M) changes in pH_i_ in the absence (**A**) or presence (**B**) of DRA_inh_-A250 (10^–5^ M) pretreatment in undifferentiated human duodenal enteroids (*n* = 31–57). (**C**) Quantitative comparison of the peak change (peak – baseline) or recovery (plateau – baseline) in pH_i_ units for enteroids exposed to CFTR_inh_-172 alone or DRA_inh_-A250 then CFTR_inh_-172. Data are expressed as means ± SEM. (**D**–**F**) Similar experiments and analyses were performed in 3-day differentiated enteroids as **A**–**C** (*n* = 37–89). Each circle represents a different cell within an enteroid. Measurements were taken from multiple enteroids across multiple coverslips. ***P < 0.001 by paired (within control or DRA_inh_-A250 groups) or unpaired, (across control or DRA_inh_-A250 groups) 2-tailed Student’s *t* test. ^+++^*P* < 0.001 compared to undifferentiated enteroids by unpaired, 2-tailed Student’s *t* test.

To investigate why CFTR inhibition increases DRA brush border expression, we examined the effect of CFTR_inh_-172 on duodenal enteroid intracellular pH (pH_i_). We hypothesized that loss of CFTR activity would lead to intracellular bicarbonate trapping (resulting in increased pH_i_) and DRA may traffic to the apical brush border as part of the cell’s effort to restore bicarbonate transport and normalize the altered pH_i_. Undifferentiated and 3-day differentiated enteroids were grown on coverslips, and CFTR_inh_-172–induced (or DMSO) changes in pH_i_ were monitored with live cell fluorescence imaging. CFTR_inh_-172 (2 × 10^–5^ M) resulted in significant increases in pH_i_ in undifferentiated and differentiated enteroids, though the slopes and magnitudes of these increases were different (Figure 7, n = 57–89, *P* < 0.001), with undifferentiated enteroids showing more rapid and greater pH_i_ changes (*P* < 0.001). DMSO (1:1,000) had no effect on pH_i_ (data not shown). Following CFTR_inh_-172, all enteroids underwent pH_i_ recovery. Pretreatment with DRA_inh_-A250 (10^–5^ M, 5 minutes) did not alter the CFTR_inh_-172–induced increase in pH_i_ but did alter the pH_i_ recovery. In undifferentiated enteroids, DRA_inh_-A250 decreased the slope and magnitude of pH_i_ recovery (Figure 7, A–C, n = 31, *P* < 0.001). In differentiated enteroids, the magnitude of pH_i_ recovery was not different with DRA_inh_-A250 (Figure 7F, n = 37, *P* = 0.213); however, the recovery slope was altered, albeit in a different way than undifferentiated enteroids (Figure 7, D and E). Thus, inhibition of CFTR function causes an increase in pH_i_, the recovery of which is at least in part influenced by DRA activity.

## Discussion

Numerous studies in animal models and humans have shown the critical importance of duodenal bicarbonate secretion in maintaining epithelial integrity and creating the appropriate intestinal intraluminal pH for digestion. In CF, with genetic loss of *CFTR*, or *Helicobacter pylori* infection, with downregulation of CFTR and PAT-1 (24), decreased duodenal mucosal bicarbonate secretion contributes to malabsorption and duodenal ulcers, respectively. While eradication of *Helicobacter pylori* with antibiotics may restore bicarbonate function, the most common way to improve small intestinal pH in CF is through chronic gastric acid suppression. Ivacaftor, a CFTR potentiator, was shown to improve proximal intestinal pH in CF patients with the G551D CFTR gating mutation (accounts for 4%–5% of *CFTR* disease-causing variants) (25). While additional CFTR modulators are FDA approved, it remains unknown whether these positively affect small intestinal pH. This gap, coupled with lack of potential bicarbonate-correcting therapy for people with CF ineligible for CFTR modulator therapies (because of genotype or medical contraindication), has driven us to seek ways to improve intestinal bicarbonate secretion independent of CFTR. Our current data are in line with prior mouse and human duodenum studies with STa (7, 11) and a recent study by Tan et el. showing that linaclotide can stimulate jejunal bicarbonate secretion in mice (9). We have shown that linaclotide stimulates both mouse and human bicarbonate secretion in the duodenum, where bicarbonate secretion is physiologically important. Furthermore, we have provided evidence for the roles of (or lack thereof) membrane trafficking of DRA and NHE3 in this process and how decreases in CFTR function may impact cellular pH.

Linaclotide represents an attractive target for restoration of deficient duodenal bicarbonate secretion in CF. Our data in both mice and humans provide greater confidence in the translatability of our results to clinical care. While we did not study other segments of the small intestine, linaclotide stimulates jejunal bicarbonate secretion in F508del CF mice (9). Braga Emidio et al. identified that linaclotide’s half-life in small intestinal fluid was 48 minutes (26), suggesting that its effect is likely to be most influential in the proximal small intestine. Comparing linaclotide-stimulated and acid-stimulated duodenal bicarbonate secretion using the same methodology (27, 28), the linaclotide effect is about 75%–100% of the stimulatory effect of acid, indicating linaclotide could produce a clinically meaningful impact for patients. With existing FDA approval, and the use of clinically relevant dosing (9), our results may provide immediate application to patients.

In the absence of CFTR, the logical targets for intestinal pH modulation are 1) increasing chloride/bicarbonate exchanger activity and/or 2) decreasing sodium/hydrogen exchange. Linaclotide increases intraluminal small intestinal fluid in mice by inhibiting NHE3-dependent sodium absorption (6, 9). Another byproduct of NHE3 inhibition is a decrease in proton secretion, which can raise intraluminal pH. Consistent with this, NHE3 inhibition in our studies “increased bicarbonate secretion,” likely by producing fewer luminal protons to neutralize secreted bicarbonate anions. In our NHE3 trafficking studies, we did not see a significant decrease in NHE3 membrane localization with linaclotide treatment. NHE3 activity may be impacted by shifting along the microvilli (23); however, our imaging resolution was unable to determine these potential nanometer shifts. Nonetheless, our in vivo data support that NHE3 inhibition alone does not account for linaclotide’s ability to increase duodenal pH. Our studies using specific DRA inhibitors and DRA protein trafficking studies in apical-out human enteroids indicate that DRA-mediated chloride/bicarbonate exchange is critical to linaclotide-stimulated duodenal bicarbonate secretion upon loss of CFTR activity. This helps explain the lack of linaclotide-stimulated I_sc_ in *Cftr*-KO mice, human biopsies with CFTR_inh_-172, and prior studies by McHugh et al. in F508del mice (6). Interestingly, we found that CFTR inhibition alone caused an increase in DRA membrane trafficking. There was also increased apical membrane DRA expression in F508del homozygous duodenal enteroids even without stimulation. In mouse intestinal crypts, Strubberg et al. identified that pH_i_ was raised in *Cftr* KO mice or WT mice treated with CFTR_inh_-172 (29). In fact, ~20 years ago, Kaunitz et al. proposed the “CF Paradox” whereby diminished bicarbonate transport may increase intracellular buffering in CF and prevent duodenal ulcer formation despite diminished bicarbonate secretion (30). Thus, we hypothesized that upon CFTR blockade (or genetic lack of *CFTR*), the rise in pH_i_ may trigger an increase in DRA expression and protein localization to at least partially restore bicarbonate secretion and limit alterations in pH_i_. Our current pH_i_ data with CFTR_inh_-172, supports this hypothesis. Bijvelds et al. reported a nearly 2,000-fold increase in *SLC26A3* mRNA in a CF ileal biopsy compared to non-CF ileum (*n* = 1 each) (31). The lack of additional membrane trafficking of DRA by linaclotide in the presence of CFTR inhibition may indicate that DRA trafficking requires CFTR function or that a ceiling effect for DRA membrane expression has already been achieved. Based on our current data, we favor the latter interpretation, since linaclotide increased DRA trafficking in F508del homozygous CF enteroids, and a comparison of MFIs across conditions showed that while the relative magnitude of DRA trafficking may differ between vehicle controls and conditions, the absolute magnitudes are similar. While CFTR inhibition caused similar pH_i_ changes in undifferentiated (crypt-like) and differentiated (villus-like) enteroids, how DRA inhibition impacted pH_i_ recovery was different. This may be due to increased expression of a larger variety of acid-base transporters in differentiated enteroids that may also contribute to pH_i_ normalization (19). Further study on how the expression and activities of DRA and other acid-base transporters are altered in CF intestine, especially in human samples, may identify new potential therapies for CF intestinal disease.

Prior to our study, our knowledge on the intestinal expression profile of DRA and other acid-base transporters was largely based on mouse studies. Wang et al. reported high expression of *Slc26a3* (DRA) RNA in the colon but negligible RNA expression in the small intestine (duodenum, jejunum, ileum). Conversely, *Slc26a6* (PAT-1) RNA expression was high in mouse duodenum, jejunum, and ileum and minimal in the colon (17). Yet, *Slc26a3*-KO mouse studies suggest a prominent role for DRA in basal and stimulated bicarbonate transport (32, 33). Human studies are sparse. Yin et al. used human duodenal enteroids to examine *SLC26A3*, *SLC26A6*, and *SLC9A3* (NHE3) mRNA expression in undifferentiated and terminally differentiated human duodenal enteroids (19). Taking advantage of published scRNA-Seq data sets from the human duodenum, we have provided a comparison on the expression of *SLC26A3*, *SLC26A6*, and *SLC9A3*, from human duodenum tissue. *SLC26A3* mRNA was generally expressed ~3–4 times higher than *SLC26A6*, *CFTR*, and *SLC9A3*. In the *CFTR*^hi^ *BEST4*^+^ cells, *CFTR* expression levels approached the level of *SLC26A3*. Of note, *SLC26A3* was also highly expressed in *BEST4*^+^ cells, though enterocytes comprise the majority of *SLC26A3*-expressing cells. These findings, put in the context of prior studies, suggest a more prominent role for DRA in the human duodenum than may have generally been appreciated from mouse studies alone. Future work examining not only the mRNA expression, but also protein expression, and especially pertinent to membrane ion transporters, membrane localization of DRA, PAT-1, and NHE3 across different segments of human intestine, may advance our knowledge regarding the regional roles of these acid-base transporters in health and disease.

A potential limitation of our study is the general reliance on pharmacologic inhibitors throughout our experiments. In *Xenopus levis* oocytes, CFTR_inh_-172 (2 × 10^–5^ M) inhibited about 70% of human CFTR current in patch-clamp experiments (34). In our experiments, CFTR_inh_-172 (2 × 10^–5^ M) significantly inhibited CFTR ion transport in mouse tissue and human enteroids, with observed results similar to those by Yin et al (19). Complementary results in *Cftr*-KO mice and CF enteroids provide further confidence in our findings with CFTR_inh_-172. For DRA, we did not perform experiments in *Slc26a3*-KO animals or cells; however, we did use 3 different DRA inhibitors, from 2 structural categories and modes of inhibition. While confidence in pharmacological inhibitors can wane with time and increased use, we believe the consistency of results across these different inhibitors provides confidence in the interpretation of the data.

In summary, we have identified that linaclotide may have more pleiotropic ion transport effects beyond CFTR- and NHE3-mediated chloride and sodium transport, respectively. We have shown, using mice, duodenal biopsies, and human duodenal enteroids, that linaclotide stimulates bicarbonate secretion and does so, at least in part, through DRA recruitment to the apical membrane. Our finding that this remains possible independent of CFTR activity or expression has potentially important implications for patients with CF, who may benefit from linaclotide’s bicarbonate-stimulatory effect, in addition to its inhibitory effect on NHE3, even if they cannot leverage its pro-chloride secretory properties (6). Furthermore, our identification of high DRA expression in the human duodenum may spur interest in identifying DRA-activating drugs targeting proximal small intestinal disease.

## Methods

### Sex as a biological variable.

Our study was performed in both male and female mice. Duodenal biopsies for direct use and for generation of enteroids were obtained from men and women. CF enteroids were generated from a person who identified as male, based on availability of the sample.

### Chemicals.

CFTR_inh_-172 was purchased from Cayman Chemical; DIDS and S3226 were purchased from MilliporeSigma. DRA inhibitors (DRA_inh_-A250, DRA_inh_-A270, DRA_inh_-4a) were synthesized and purified as previously described (15, 16, 35). Linaclotide was purchased from Santa Cruz Biotechnology.

### Animals.

Adult (8- to 16-week-old) C57BL/6J mice from The Jackson Laboratory were used as WT control mice. *Cftr*^tm1Unc^-KO mice were obtained from Case Western University CF Mouse Models Core Facility and maintained on 50% PEG-3350 with electrolyte solution in their drinking water with standard chow.

### In vivo measurement of bicarbonate secretion.

In vivo measurement of duodenal bicarbonate secretion was performed using a well-validated technique (36). Animals were maintained with free access to food and water for up to 1 hour before the experiment, then fasted from chow. Mice were anesthetized with oxygen-delivered isoflurane (1%–3%) at 1 L/min via a vaporizer (Braintree Scientific, Inc). Mouse temperature was monitored by rectal probe and maintained at 37°C through automated warming using a controlled warming pad (ATCC 2000, World Precision Instruments). Anesthetic plane was assessed by respiratory rate and toe pinch reflex. Respiratory and heart rates were determined every 15 minutes. Animals could be sustained for more than 3 hours under these experimental conditions.

To isolate the proximal duodenum, the abdomen was opened by a central vertical incision, and the proximal 5–10 mm of duodenum, from the pylorus to just proximal to the entry of the common bile duct, was isolated in situ without compromising vascular supply. A small polyethylene tube (PE-50) with a distal flange was advanced to the duodenal bulb via the stomach, and a ligature was secured around the pylorus. A distal intestinal incision was made, and PE-50 flanged tubing was advanced to just proximal to the entry of the pancreaticobiliary duct to prevent entry of the pancreatic or biliary secretions and allow for collection of effluent from isolated segment. The isolated duodenal segment was gently flushed and then continuously perfused (Harvard Infusion Pump, Harvard Apparatus) at a rate of 0.21 mL/min with 154 mM NaCl (37°C) ± stimulatory/inhibitor drugs. Effluents from the isolated segment were visually free of bile and blood throughout all experiments. After an initial 15-minute washout period, basal bicarbonate secretion (with luminal saline perfusion) was measured for 30 minutes. Subsequently, linaclotide (10^–9^ M, 10^–7^ M, 10^–5^ M) ± DRA_inh_-A250 inhibitor (10^–5^ M), CFTR_inh_-172 (2 × 10^–5^ M), or S3226 (10^–5^ M) was perfused intraluminally for 30 minutes. When multiple doses of drugs were studied, they were perfused sequentially in the same animals. These doses were selected based on previously published data and similarities with clinical dosing. Forskolin (10^–4^ M) ± CFTR_inh_-172 (2 × 10^–5^ M) was perfused intraluminally for 45 minutes. After each experiment, the length of the duodenal test segment was measured in situ to the nearest 0.5 mm.

Sample volumes were measured by weight to the nearest 0.01 mg. The amount of bicarbonate in the effluents was quantitated by a validated micro back-titration method (36). Briefly, 100 μL of 5 × 10^–2^ M HCl was added to 2 mL sample with 2 mL of double-distilled water. Samples were then gassed with N_2_, prewashed in Ba(OH)_2_ to remove all CO_2_, and back-titrated with 2.5 × 10^–2^ M NaOH to an endpoint of pH 7.0 using an automated pH meter/titration unit (TIM 856 Radiometer). Bicarbonate secretion was determined in 15-minute periods and expressed as micromoles per centimeter per hour and presented as bicarbonate output over time or net peak bicarbonate output (peak output minus average basal).

### In vitro measurement of ion transport and bicarbonate secretion.

For murine measurements, mice were anesthetized as above, and the proximal 1 cm of duodenum was removed and placed in ice-cold 3 × 10^–1^ M mannitol solution with indomethacin (10^–5^ M) to prevent endogenous prostaglandin formation. The mesentery was dissected off and the duodenum was opened along the mesenteric edge. The muscular layer was scraped from the mucosa using a glass slide while being bathed in the indomethacin-containing solution. Stripped duodenal mucosae were mounted between two 0.1 cm^2^ aperture sliders placed in temperature-controlled Ussing chambers maintained at 37°C (P2300, Physiologic Instruments). For human endoscopic biopsy measurements, biopsies were collected in the same iso-osmolar mannitol solution with indomethacin and then mounted directly between two 0.031 cm^2^ aperture sliders. Tissue was bathed in apical solutions containing (in mM) NaCl 115, K-Gluconate 5.2, Na-Gluconate 25, MgCl_2_ 1.2, CaCl_2_ 1.2, mannitol 10, gassed with 100% O_2_, and basolateral solutions containing (in mM) NaCl 115, K_2_HPO_4_ 2.4, KH_2_PO_4_ 0.4, NaHCO_3_ 25, MgCl_2_ 1.2, CaCl_2_ 1.2, glucose 10, gassed with 95% O_2_/5% CO_2_. Ion transport was measured by I_sc_, where tissues were voltage-clamped at 0 mV using a voltage-clamp apparatus (VCC600, Physiologic Instruments). To monitor transepithelial resistance, the voltage-clamp was released, and the open circuit voltage was recorded every 10 minutes. Resistance was calculated using Ohm’s Law. To measure bicarbonate secretion, we utilized the automatic pH-stat method, as previously described (7, 37). In brief, a pH microelectrode was placed in the apical bath and set at the pH of the unbuffered apical solution (typically ~6.8). The titrator (Titrando 902, MetrOhm) was set to titrate 5 × 10^–3^ M HCl in 0.2 μL aliquots as the pH of the apical bath rose above the pH set point. Tiamo software (MetrOhm) was used to control the rate of titration and continuously measure the amount of HCl titrated into the apical bath and the apical bath pH. The setup as described allows for simultaneous measurement of bicarbonate secretion and I_sc_ without electrical interference. Bicarbonate secretory rates (μmol/cm^2^•h) were calculated in 5-minute intervals by noting the amount titrated, the concentration of titrant, and the surface area of the slider aperture. In a subset of experiments, forskolin (10^–4^ M, serosal) was added at the end of the experiment to assess tissue viability.

### Measurement of pH_i_.

pH_i_ levels were measured via epifluorescence microscopy on a Leica Thunder DMi8 imaging system. Enteroids were grown on collagen-coated 18 mm coverslips in WENR (undifferentiated) alone or changed to EN for 3 days (differentiated) after initial seeding in WENR. On day of experiment, enteroids were incubated with the cell-permeant ratiometric pH indicator SNARF-5F 5-(and-6)-carboxylic acid, acetoxymethyl ester, acetate (5 μM) (Thermo Fisher Scientific), for 30 minutes at room temperature. Following a 15-minute wash period, imaging experiments were performed in physiologic buffer (in mM: NaCl 115, K_2_HPO_4_ 2.4, KH_2_PO_4_ 0.4, MgCl_2_ 1.2, CaCl_2_ 1.2, HEPES 10, glucose 10). Coverslips were imaged in a heat-controlled (37°C), gassed (95% O_2_/5% CO_2_) enclosure. Using LasX software, regions of interest (ROIs) were selected by encircling visualized cells for ratiometric measurements within each ROI. Measurements were taken every 5 seconds. Baseline pH_i_ was recorded for 2–3 minutes, followed by application of CFTR_inh_-172 (20 × 10^–5^ M) or DRA_inh_-A250 (10^–5^ M, 5 minutes), then CFTR_inh_-172. Upon full pH_i_ recovery (typically 4–6 minutes), pH calibration was performed using serial addition of nigericin + valinomycin in pH 4.5–7.5 buffers (Thermo Fisher Scientific) for at least 5 minutes each.

### Single-cell sequencing analysis.

The R toolkit Seurat was used to conduct an analysis and quality control of scRNA-Seq data from existing literature (38). We clustered the cells according to the Seurat default settings. In brief, we embedded the cells through a K-nearest neighbor graph, reduced the data with principal component analysis, and applied the Louvain algorithm for clustering. We visualized these clusters with uniform manifold approximation and projection. To annotate major cell types and compare gene expression in crypts versus villi, we used the metadata annotations provided by Elmentaite et al. (21) and Busslinger et al. (20), respectively. We used violin and feature plots to display gene expression profiles and FeatureScatter to compare the coexpression of 2 genes by cell type.

### Human duodenal organoids.

Duodenal organoids were established and cultured as previously described (39). In short, crypt cells were isolated from duodenal endoscopic biopsies using a series of PBS washes followed by incubation with cold chelation buffer (distilled water with 5.6 mM Na_2_HPO_4_, 8.0 mM KH_2_PO_4_, 96.2 mM NaCl, 1.6 mM KCl, 43.4 mM sucrose, 54.9 mM d-sorbitol, 1 mM dl-dithiothreitol) with 2 μM EDTA for 30 minutes on ice. After incubation, EDTA was removed, and the biopsies were vigorously resuspended in cold chelation buffer (without EDTA). The resuspension was allowed to sit on ice for 1 minute to allow biopsies to settle by gravity. The supernatant was collected. This process was repeated 3–5 times until no more crypts were present in the supernatant. The crypt-containing supernatants were pooled and centrifuged at 600*g* for 5 minutes, followed by resuspension in 40 μL basement membrane extract/dome (BME, Culterex Pathclear Type 2 BME) and submerged in WENR complete media (DMEM/F12, 1 mM HEPES, 1× Glutamax, 1 mM N-Acetylcysteine, 1× B-27, 0.5 μM A83-01, 1× Penicillin-Streptomycin-Glutamine, 10 mM gastrin, 10 μM SB-202190, 50 ng/mL EGF, 10 mM nicotinamide, 1× normocin) with Rho-associated kinase inhibitor (Y-27632 10 μM, MilliporeSigma) and CHIR99021 (10μM, MilliporeSigma) for the first 2 days, followed by WENR complete media only until ready to passage. Duodenal enteroids were passaged by disrupting BME with cold (4°C) media, and 1× cold (4°C) PBS (without Ca^2+^ and Mg^2+^), then centrifuging at 600*g* for 3 minutes at 4°C. Supernatant was aspirated and single cells were isolated by adding warm (37°C) TrypLE Express (Gibco, Thermo Fisher Scientific) and were incubated for 10 minutes at 37°C. An equal volume of FBS was added to quench trypsin, and cells were centrifuged at room temperature for 5 minutes at 600*g*. The pellet was resolubilized in BME (40 μL/dome) and plated in a 24-well tissue culture plate. Generation of apical-out enteroids was performed as previously described (22). Briefly, enteroids were dislodged from BME using with 1× cold (4°C) PBS (without Ca^2+^ and Mg^2+^) and resuspended in 5 mM EDTA in PBS for 1 hour at 4°C. Enteroids were then centrifuged at 200*g* for 3 minutes and resuspended in WENR or EN media (WENR without Wnt and R-Spondin) and seeded in ultra-low-attachment 24-well plates (Corning Costar, Thermo Fisher Scientific) for 3 days. On day 3, undifferentiated and/or differentiated enteroids were harvested for experiments. Anjaparavanda Naren and Kyu Shik Mun (Cedars-Sinai, Los Angeles, California, USA) provided CF enteroids.

### QPCR.

Quantification of mRNA transcript levels in undifferentiated and differentiated apical-out enteroids was performed. Briefly, total RNA was extracted from fresh apical-out enteroids using Zymo Research Direct-Zol RNA miniprep kit according to the manufacturer’s instructions. cDNA was synthesized from 1 to 2 μg of RNA using High-Capacity RNA-to-cDNA Kit (Zymo Research). QPCR was performed on a StepOne Plus real-time PCR system (Applied Biosystems, Thermo Fisher Scientific) using PowerUP SYBR Green Master Mix (Applied Biosystems, Thermo Fisher Scientific). Each sample was run in triplicate in a 20 μL reaction volume containing 5 ng cDNA/reaction. PCR was carried out for 40 cycles at 95°C (denature) for 30 seconds, and 60°C (anneal/extend) for 30 seconds, and amplification data were collected after each cycle. The sequences of optimized gene-specific primers are listed in Supplemental Table 1. *GAPDH* was used as the reference gene to obtain ΔCt values. The relative expression levels of the target genes were calculated using the 2^-ΔΔCt^ method.

### Confocal microscopy.

Treated day 3 apical-out enteroids were fixed in 2% paraformaldehyde immediately after treatment and stored at 4°C until processing. All steps were performed in ultralow-attachment plates. Fixed enteroids were permeabilized for 30 minutes with permeabilization buffer (3% BSA, Thermo Fisher Scientific), 0.5% Triton X-100, and 0.7% Saponin (MilliporeSigma) in PBS and blocked for 60 minutes with gentle shaking in blocking buffer (3% BSA, 0.2% Triton X-100). Samples were incubated overnight at 4°C with primary antibodies (Supplemental Table 2) diluted in antibody diluent (3% BSA, 0.2% Triton X-100, and 0.5% Saponin in PBS), washed 4 times for 30 minutes each in antibody diluent buffer, and incubated with secondary antibodies (Supplemental Table 2) for 1 hour with gentle shaking at room temperature. Samples were washed 4 times for 30 minutes each in antibody diluent buffer. Samples were then carefully resuspended in SlowFade gold antifade mounting medium (Thermo Fisher Scientific), mounted on microscope slides, and sealed with glass coverslips (1.0 mm) (Thermo Fisher Scientific). Slides were imaged using Leica Stellaris 8 Confocal microscope. *Z*-stacks of 0.33 μm sections/sample were obtained and analyzed using FIJI Software (ImageJ, NIH). Following acquisition, image settings were adjusted to optimize the signal/noise ratio. For quantitative analysis, the same settings were used across the different conditions compared, and the protein-of-interest MFI after background subtractions was analyzed. Villin was used as a marker for the plasma membrane and used to identify the area for fluorescence intensity measurements. Drug-treated MFI measurements were normalized to corresponding vehicle control MFI measurements.

### Statistics.

Results are expressed as means ± SEM for a series of *n* experiments. Statistical analysis was performed by using the 2-tailed Student’s *t* test for unpaired data or by 1-way ANOVA with repeated measures analysis, as appropriate. A *P* value less than 0.05 was considered significant.

### Study approval.

All animal protocols were approved by the Institutional Animal Care and Use Committee at Stanford University (no. 33183). Children and adults undergoing upper endoscopy for clinical indications were approached to obtain additional duodenal biopsies for research under an Institutional Review Board–approved protocol (Stanford University, no. 47536). Participants without endoscopic and microscopic evidence of duodenal pathology were included in this study.

### Data availability.

The data supporting the findings of this study are available within the paper and its supplement. For graphs not including actual data points, data used to generate figures are available in the Supporting Data Values file.

## Author contributions

JBS performed and analyzed experiments and drafted, edited, and approved the final manuscript. AMT performed and analyzed experiments and drafted and approved the final manuscript. SMA performed and analyzed experiments and drafted and approved the final manuscript. VVU performed and analyzed experiments and drafted and approved the final manuscript. JEC performed and analyzed experiments and approved the final manuscript. YF performed and analyzed experiments and approved the final manuscript. JG performed and analyzed experiments and approved the final manuscript. MOA contributed technical expertise and material support and approved the final manuscript. OC contributed technical expertise and material support and approved the final manuscript. CJK contributed technical expertise and material support and edited and approved the final manuscript. ZMS contributed study conceptualization, experimental design, and funding and drafted, edited, and approved the final manuscript.

## Supplementary Material

Supplemental data

Supporting data values

## Version 1 06/13/2024

In-Press Preview

## Version 2 07/22/2024

Electronic publication
