# Supplementary material for: DRA involvement in linaclotide-stimulated bicarbonate secretion during loss of CFTR function
Source: JCI Insight. 2024 Jun 13;9(14):e172364. doi: 10.1172/jci.insight.172364 (PMC11383163; doi:10.1172/jci.insight.172364)
Supplement: Supplemental data [file jciinsight-9-172364-s035.pdf]

SUPPLEMENTARY TABLES AND FIGURES

Supplementary Table 1. qPCR gene-specific Primer sequences

Gene	Forward Primer (5′-3′)	Reverse Primer (5′-3′)
SLC26A3	CAGCCCCCTATTACACCTGA	CCTCCTGTGCTCTCCTGAAC
SLC26A6	GCCTTGAACGACTCCATGAT	TGTGAGACGAAGACCTGCAC
CFTR	CCTATGACCCGGATAACAAGGA	GAACACGGCTTGACAGCTTTA
SLC9A3	CCTGACCATCAAGCCTCTGG	ACATTCAGGATCCGGTCTCG
LGR5	GATGTTGCTCAGGGTGGACT	GGGAGCAGCTGACTGATGTT
GUCY2C	GGCTGTCCTTTAGTTCCCAGG	GAAAGTAGCGTTCACAGTCACAT
MYO6	TAACCCACTCCTAGAAGCCTTT	GCACCAGCACACAACCTATAA
GAPDH	GAAGGTGAAGGTCGGAGTC	GAAGATGGTGATGGGATTTC

SLC26A3 (down-regulated in adenoma, DRA), *SLC26A6* (putative anion transporter-1, PAT-1), *CFTR* (cystic fibrosis transmembrane conductance regulator), *SLC9A3* (sodium/hydrogen exchanger 3, NHE3), *LGR5* (leucine rich repeat containing G protein-coupled receptor 5), *GUCY2C* (guanylate cyclase 2C), *MYO6* (myosin VI), *GAPDH* (glyceraldehyde-3-phosphate dehydrogenase)

Supplementary Table 2. List of antibodies

Primary Antibodies	Dilution	Manufacturer
**DRA (mouse)**	1:200	Santa Cruz (sc-376187)
**NHE3 (rabbit)**	1:400	Novus Biologicals (NBP1-82574)
**Villin (rabbit)**	1:400	ThermoFisher (PA5-29078)
**Villin (mouse) 1D2C3**	1:200	Santa Cruz (sc-58897)
**Hoechst 33342**	Per manufacturer’s protocol	Abcam (ab228551)

Secondary antibodies	Dilution	Manufacturer
**Goat Anti-Rabbit Alexa flour 594**	1:300	Abcam (ab150088)
**Goat Anti-Mouse Alexa flour 647**	1:300	Abcam (ab150115)
**Goat Anti-Mouse IgG H&L (Alexa Fluor® 488)**	1:1000	Abcam (ab150113)

SUPPLEMENTARY FIGURES

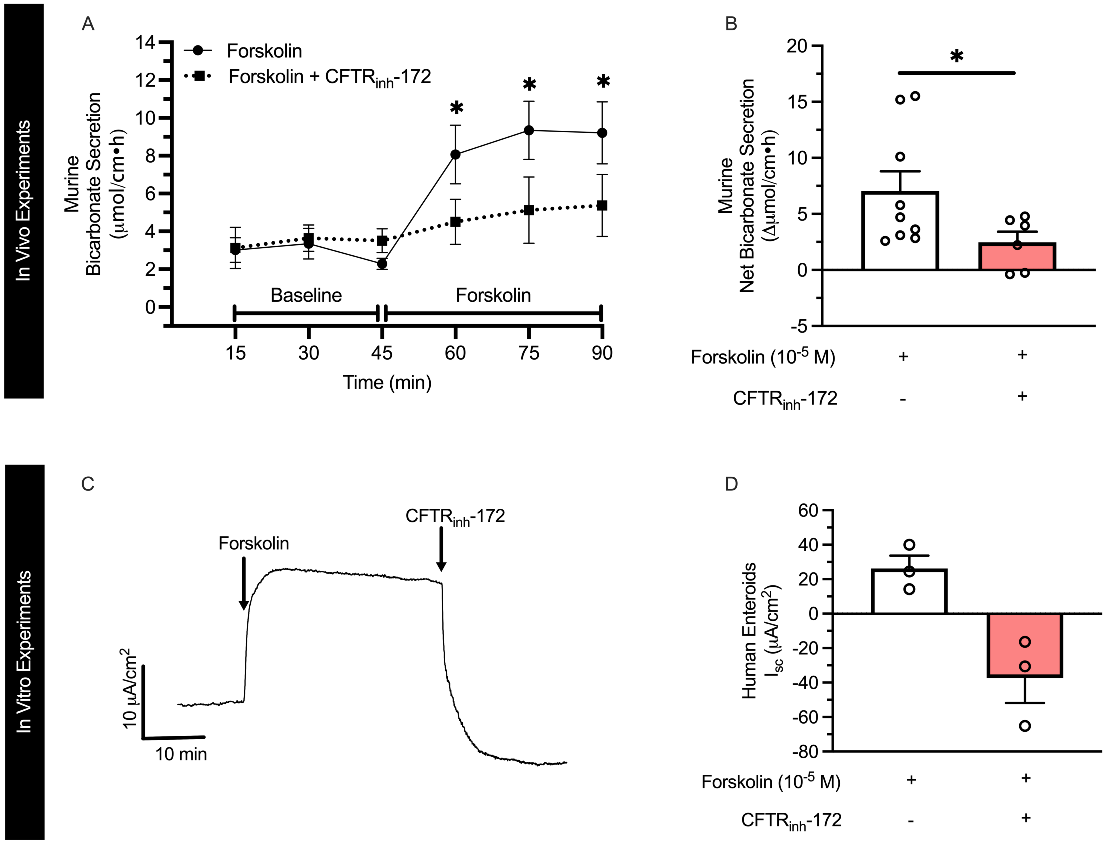

Supplemental Figure 1. CFTR$_{inh}$-172 inhibits forskolin-stimulated duodenal bicarbonate secretion in mice and human enteroids. A. To compare the effect of pharmacologic CFTR inhibition with CFTR$_{inh}$-172 on linaclotide and forskolin-stimulated bicarbonate secretion, we performed similar in vivo experiments as Figure 2A. Duodenum of wildtype mice were perfused with saline then forskolin (10^{-4} M) (black circles) or saline + CFTR$_{inh}$-172 (2×10^{-5} M) then forskolin (10^{-4} M) (black squares). Bicarbonate secretion was calculated from the perfusates similar to Figure 2A. *, $P<0.05$ by one-way ANOVA. **B.** Comparison of net change in forskolin-stimulated bicarbonate secretion (peak response – baseline secretion) in the presence or absence of CFTR$_{inh}$-172. Columns with whiskers represent mean ± SEM with each circle representing a different experiment. Mean ± SEM percent inhibition indicated above columns. *, $P<0.05$ by unpaired two-tailed Student's t-test. **C.** Representative trace of forskolin (10^{-5} M, basolateral)-stimulated short-circuit current, followed by inhibition by CFTR$_{inh}$-172 (2×10^{-5} M, basolateral), in undifferentiated human duodenal enteroids monolayers. **D.** Quantification of replicate experiments performed in C. Columns with whiskers represent mean ± SEM with each circle representing a different experiment.

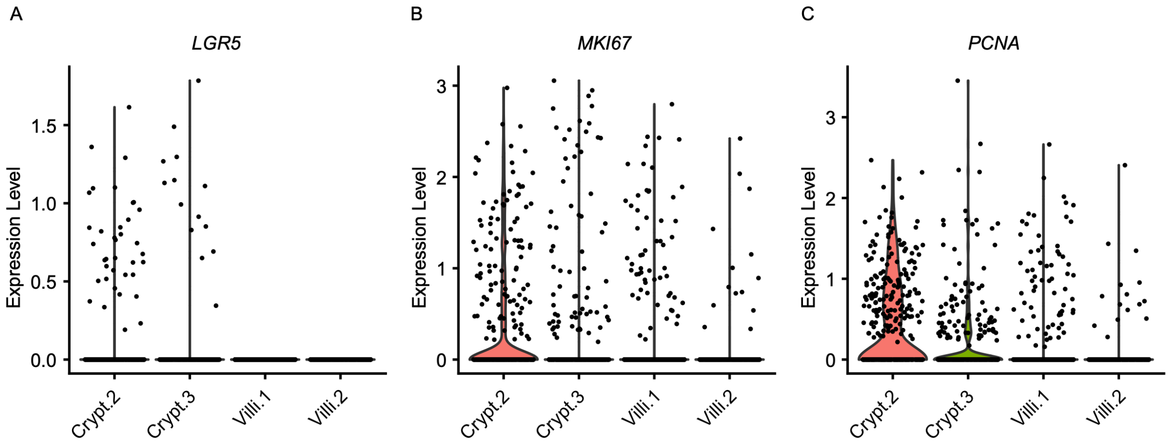

Supplementary Figure 2. Validation of previously published human duodenum scRNA-seq datasets. A-C. To verify the crypt vs. villi identity in the Busslinger et al. dataset we examined the expression of *LGR5* **(A)**, *MKI67* **(B)**, and *PCNA* **(C)**.

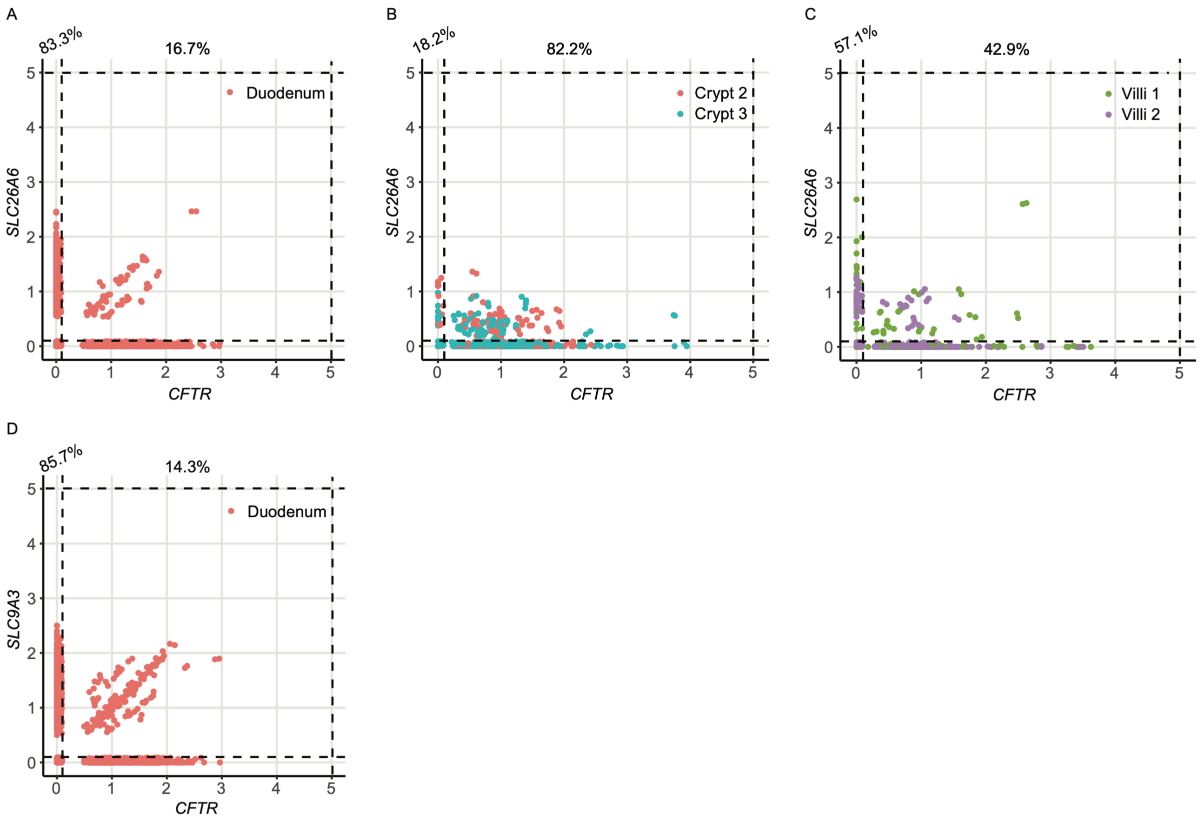

Supplementary Figure 3. Co-expression of *CFTR* and *SLC26A6* (PAT-1) and *SLC9A3* (NHE3) from human duodenal sc-RNAseq data. A-C. Co-expression of *SLC26A6* (PAT-1) and *CFTR* mRNA using FeatureScatter based on Elmentaite et al.[14] enterocytes (A) and Busslinger et al.[15] crypt and villi (B and C) datasets. **D.** Co-expression of *SLC9A3* (NHE3) and *CFTR* mRNA using FeatureScatter based on Elmentaite et al. enterocytes. There were insufficient numbers of *SLC9A3* positive cells in the Busslinger et al. dataset to do the same analysis as B and C. Numbers on top of graphs represent the percentage of *SLC26A6*- or *SLC9A3*-expressing cells that express these only (left) or *SLC26A6/SLC9A3* and *CFTR* (right).

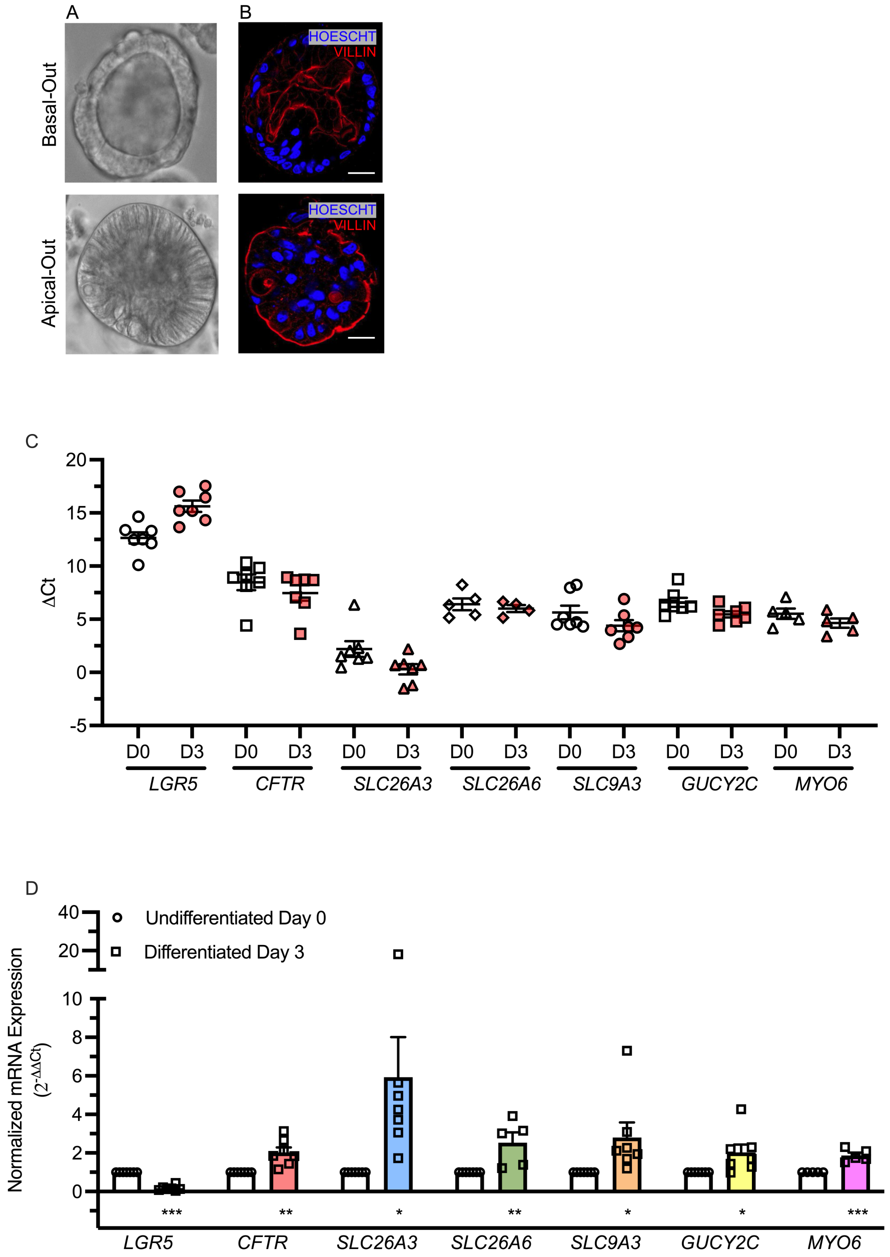

Supplementary Figure 4. Characterization of Apical-Out Duodenal Enteroids. A. Sample brightfield microscope images of basal-out (top) and apical-out (bottom) human duodenal enteroids. **B.** Representative confocal immunofluorescent images of basal-out (top) and apical-out (bottom) human duodenal enteroids stained with villin (apical membrane) and Hoescht (cell nucleus). Scale bar = 20 μm. **C and D.** mRNA expression of *LGR5, CFTR, SLC26A3, SLC26A6, SLC9A3, GUCY2C, and MYO6* in undifferentiated and differentiated (x3 days) apical-out human duodenal enteroids. Delta CT was calculated by comparing to *GAPDH* expression (C) and Delta Delta CT was calculated by comparing Differentiated Day 3 enteroids to Undifferentiated Day 0 enteroids. *, P<0.05; **, P<0.01; ***, P<0.001 compared to Undifferentiated Day 0 enteroids by unpaired two-tailed Student's t-test.

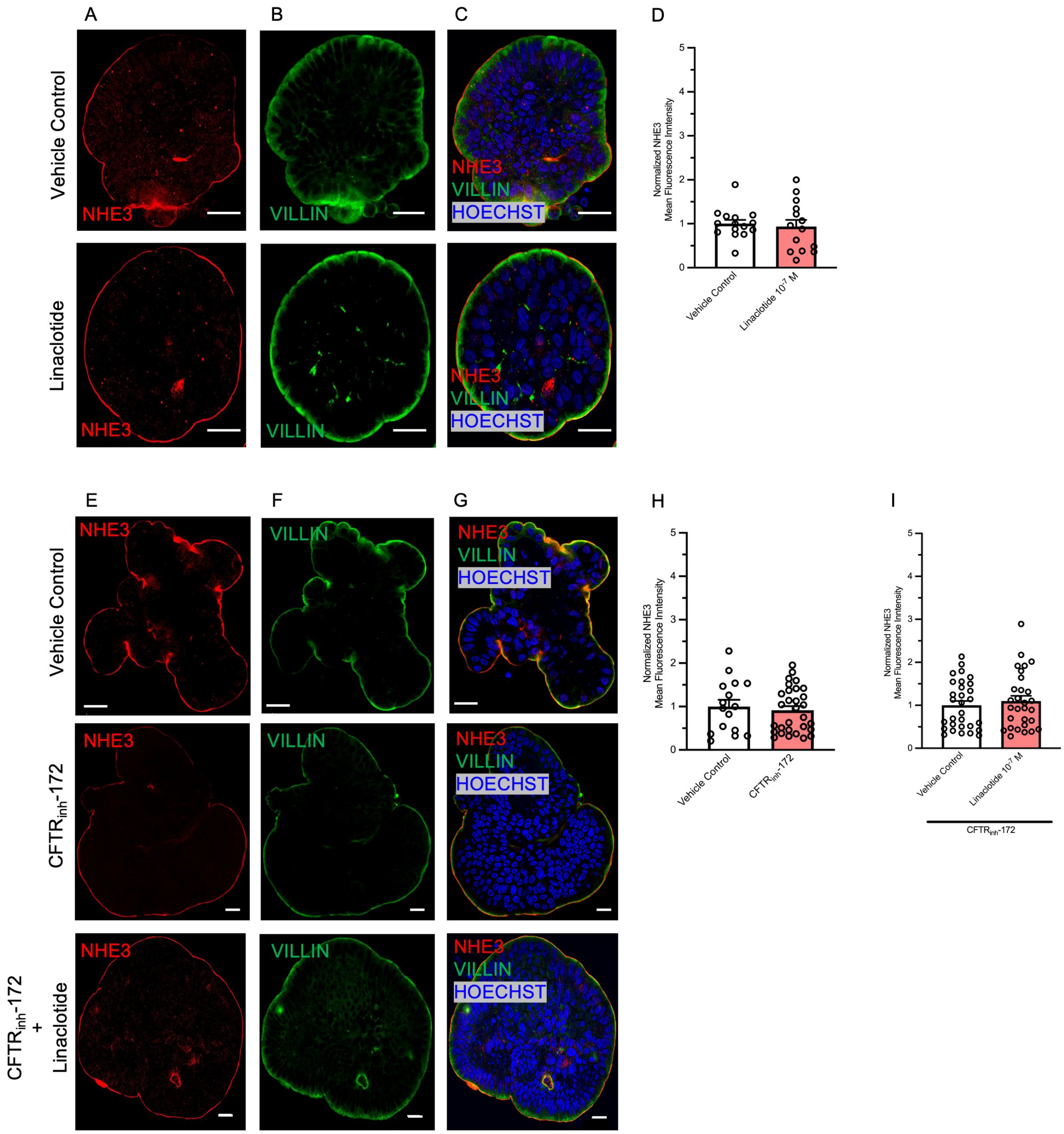

Supplemental Figure 5. Linaclotide does not change the membrane expression of NHE3 in apical-out human duodenal enteroids. A-C. Representative confocal microscopy images of NHE3 (A), villin (B), and NHE3 and villin and Hoescht (C) during control conditions (water, 40 minutes, top) or linaclotide (10^{-7} M, 40 minutes, bottom). **D.** Quantification of NHE3 present at the apical membrane using villin to define apical membrane, similar to Fig. 6. **E-G.** Representative images for apical membrane NHE3 mean fluorescence intensity following CFTR$_{inh}$-172 (2×10^{-5} M, 40 minutes) with or without linaclotide (10^{-7} M, 40 minutes) treatment. **H-I.** Quantification of images, normalized to vehicle controls for comparison. Vehicle control for linaclotide was water and for CFTR$_{inh}$-172 was DMSO. Columns with whiskers are mean ± SEM with each dot representing a different enteroid. Enteroids from three different patients were used for each condition. Significance determined by unpaired two-tailed Student's t-test. For enteroids treated with both CFTR$_{inh}$-172 and linaclotide, enteroids were pretreated with CFTR$_{inh}$-172 for 40 minutes prior to linaclotide treatment.
